# Parameter Study on Friction Surfacing of AISI316Ti Stainless Steel over EN8 Carbon Steel and Its Effect on Coating Dimensions and Bond Strength

**DOI:** 10.3390/ma14174967

**Published:** 2021-08-31

**Authors:** George S. N. Rethnam, Subramanian Manivel, Vijay K. Sharma, Chidurala Srinivas, Asif Afzal, Abdul Razak R.K., Sagr Alamri, C. Ahamed Saleel

**Affiliations:** 1 Department of Mechanical Engineering, St. Joseph’s College of Engineering, Chennai 600119, India; gsnixons@gmail.com; 2 Department of Physics, Shyam Lal College, University of Delhi, New Delhi 110032, India; vasuvijay2001@yahoo.com; 3 Department of Mechanical Engineering, Vaageswari College of Engineering, Telengana 505481, India; chiduralasrinivas@gmail.com; 4 Department of Mechanical Engineering, P. A. College of Engineering, (Affiliated to Visvesvaraya Technological University, Belagavi), Mangaluru 574153, India; abdulkaladgi@gmail.com; 5 Department of Mechanical Engineering, School of Technology, Glocal University, Delhi-Yamunotri Marg, SH-57, Mirzapur Pole, Saharanpur District, Uttar Pradesh 247121, India; 6 Department of Mechanical Engineering, College of Engineering, King Khalid University, Abha 61421, Saudi Arabia; salamri@kku.edu.sa (S.A.); ahamedsaleel@gmail.com (C.A.S.); 7 Department of Mechanical Engineering, The University of Akron, Akron, OH 44325-3903, USA

**Keywords:** friction surfacing, AISI316Ti stainless steel over EN8 carbon steel, response surface methodology, bond strength, depth of coating and coating width

## Abstract

Friction surfacing is a solid-state coating process that uses plastic deformation to improve the efficiency of the core metallic pattern, resulting in fine-grained coatings with superior wear and corrosion properties. This article focuses on the development of inherently homogeneous, non-diluted coating of AISI316Ti stainless steel above EN8 and also encloses the empirical relationship for the prediction of bond strength (B_s_), coating thickness (C_t_), and coating width (C_w_). The key individualities for bonding geometry were believed to be the process parameters such as rotational speed (rpm), traverse speed (mm/s), and axial load (kN). The effect of input parameters on the bond’s external dimensions and strength was investigated using a multi-objective optimization approach through experimentation. The bond’s strength improved as the coating thickness was reduced and the coating width was increased. The grain-refined coatings superimposing martensitic microstructure with no deposition of carbide particles added value to the metallurgical study using the scanning electron microscope.

## 1. Introduction

As a solid-state process for creating corrosion-resistant and hard-facing surfaces that increase the efficiency of primary metal patterns, friction surfacing has been essentially inevitable in recent periods. In modern days, friction surfacing has revived interest, consumed by the need for superior overcoat solutions. Increased results in friction processing have led to new concerns for researchers in the field. During the last few years, friction surfacing has received a lot of attention. In the recent past, as it relates to the reclamation of worn components, it has been shown to be effective in the rebuilding of worn-out shafts [1]. The heat produced yields a visco-elastic layer that results in a substrate-material bond. The processing of these surfaces without any dilution distinguishes this technique from other categories of surface modification processes [2]. Friction surfacing on varied substrates and coating strong coatings over soft substrates combinations over hard surfaces, as well as soft coatings [3,4].

**Figure 1:**
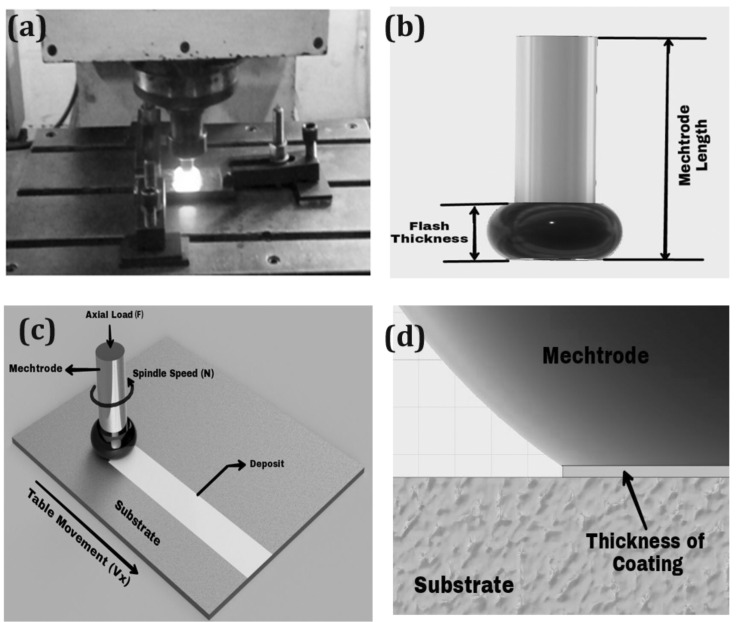
Friction surfacing and its dimensional measurements (**a**) during friction surfacing. (**b**) Mechtrode. (**c**) Friction surfacing process. (**d**) Coating thickness.

During this friction surface cycle, the rod is rotated at a precise speed and uninterruptedly fed to the plate with the load perpendicular to the axis acting on the rod as shown in Figure 1b. The cycle begins with a rotating consumable rod (mechtrode) in contradiction of a substratum under rotational speed, variable axial load, and speed of traversal. However, there are few accounts of friction surface modification and even fewer examples of it in practice. In most coating procedures for mending castings, arc or gas welding is also used [5,6]. In most coating procedures for mending castings, arc or gas welding is also used. Material flow over the contact area arises because of the constant motion of the process parameters designated above. When the plate of the substratum moves at a particular point, the metal that has undergone plasticization sheds over it. The thickness and width of the coating are solely determined by the process parameters as shown in Figure 1a,c,d. As a result, when process parameters are changed, there is no way to determine the accuracy and sensitivity [7]. The goal of this experiment was to use conventional techniques to relate the main process parametric linkages to friction surfacing (FS) with the addition of inductive heating [8].

The key need to use this method is to repair the surfaces. There are many approaches for renovating the damaged surfaces which are too expensive and intricate. Thus, they industrialized this practice for economic purposes and to give an adequate coating over the damaged surface [9,10]. Many divergent combinations can cover innumerable kinds of materials in this process. A friction surfacing process was used to coat EN8 carbon steel with AISI316 stainless steel in an attempt to prevent corrosion. Process variables such as traverse speed, axial load, and rotating speed have all had a significant impact on coating thickness. This method strongly depends on the effect of friction surfacing parameters such as axial load, rotational speed, and traverse speed [9]. Some researchers reported the impact of the above parameters by building a decision model to improve bonding strength, coating thickness, and coating width. The intensification of axial load increased the bond’s strength, condensing the coating thickness. The compromised area decreased at higher axial loads and slowly increased at higher traverse speeds [11,12,13]. Based on corrosion performance research on austenitic stainless steel deposited over mild steel, Friction Surfacing (FS) is used in the construction of chemical pumps and petrochemical pressure vessels [14]; the use of a metallic adhesive to join the coating to the body boundaries of the push-off the maximum load that may be applied to the adhesive’s tensile strength [15]. 

Friction surfacing develops the difficulty of the joining mechanism that requires temperature activity and joining. Along the mechtrode, which defines the visco-elastic plasticized region stage, frictional heat is generated, which typically occurs in the center of the covering. The temperature of the process between the substrate and the thread is influenced by frictional force. In determining coating consistency and geometry, process temperature plays a key role. The wider coating width (C_w_) and the minimum coating thickness (Ct) are obtained for an increase in process temperature and axial load. The reduction in rotational speed has affected the temperature of the process, resulting in thick deposits [16]. Because the deposited metal diameter is less than the pin diameter, the consumable rod is plasticized in the middle and not entirely transferred to the edges when sufficient heat is generated owing to friction in the interface. The slippage between the spinning consumable rod and the deposited layer causes friction Surfacing (FS) to be transmitted along a rotational contact plane [17,18]. So, after that, with the help of axial force, the interface consists of partial solid and partial liquid metal form, and then the metal begins to settle over the substrate. Since the low hardness of the zone over the bonding occurs, the coating may have a fine-grain microstructure due to rotating movement and mechtrode forging action. The ductile coating characteristic would be greater than the mechtrode’s [19]. The granular microstructure and inter-metallic growth worsen the consistency of the coating, which gives the friction surface certain advantages. No defects are observed during this process such as surface cracks, porosity, or inclusion of slag. In comparison, the absence of noxious gases and the non-release of emissions of radioactivity makes this device more eco-friendly as opposed to other solid-state processes. By creating dynamically recrystallized material, significant plastic deformation occurred on the coated material, resulting in the production of a suitable bonding geometry zone as a result of rotational speed [20,21]. Response Surface Methodology (RSM) creates an approximate figure for the analytical relationship between the independent factors and the response variables, according to several studies, which helps elucidate the peculiarities of coating measures [22,23,24,25]. The goal of this paper is to analyze and refine the friction surfacing parameters such as traverse speed, rotational speed, and axial load with the coating dimensions and bond strength. Using a response surface methodology, an empirical model is produced to obtain the optimal output for the friction surfacing of AISI316Ti over EN8 alloy in which a comparative analysis was not previously performed in the literature research.

## 2. Materials and Methods

AISI316Ti stainless steel as a mechtrode (10 mm rod diameter × 30 mm rod length) and EN8 as a substratum (10 mm thick plate) are the materials used in this study, which is the most widely used of all stainless steels. Its chemical composition, mechanical properties, and corrosion resistance give stainless steel more potential than cost and mass density at a comparatively lower value. It can be used for long periods at elevated temperatures without losing its corrosion resistance. At high temperatures, these AISI316Ti stainless steel rods can be used. This resistance is fine for sulfuric acids, chlorinated acids, and sulfate acids. As part of a few undertakings, including the brewing, chemical, marine, and pharmaceutical sectors, AISI316Ti bars are included. EN8 has a very good weldable property and has a lightweight and rigid case, which for carburized components is considered to be the best steel. Enhanced ductility, strength, higher mechanical properties, and better friction surfacing properties are provided by EN8. It is one of the worldwide commercial commodities used because of its excellent formability in the plastic process and the flatness of the material which are often used in the marine industry. In preparation for being austenized and then tempered, this steel may be heat treated. Table 1 and Table 2 demonstrate the chemical composition of the AISI316Ti stainless steel mechtrode and EN8 carbon steel substrate, as determined by X-ray fluorescence spectroscopy (PW2404).

As the mechtrode and substrate, stainless steel plates AISI316Ti and EN8 are employed. The surface flatness was machined by milling at the top and bottom of the rod, and the surface finish was rendered by a grinding machine to achieve an oxide-free level surface. Before the experiment, mechtrode and substrate were washed with acetone to eliminate pollution. To precede the experimental work, a customized friction-surfacing machine (Modified milling machine JMD-18 JET) was used. With a nominal rotational speed of 3000 rpm, the system can be loaded up to a maximum axial load of 10 kN.

Safety measures have been taken to prevent any object from being trapped during surfacing. Method parameters such as the rotational speed (rpm) of the mechtrode spindle (A), the traverse velocity of the substrate (mm/s) (B), and the axial load (kN) of the mechtrode (C) are calculated by test experiments. Reachable method parameter limits are selected in such a manner that any visible defects can be excluded from the bond. Surface friction was performed in accordance with the conditions shown in Table 3. By integrating several input parameters, RSM is a lively approach used to optimize process parameters and it helps to determine the relationships and performance of response parameters [26]. Based on introductive examinations, the levels of the criteria are chosen. In a generalized equation, the response surface is expressed as [27]

$$y=\beta_{0}+\sum_{i=1}^{s}\beta_{i}x_{i}+\sum_{i=1}^{s}\beta_{ii}x_{i}^{2}+\sum_{i=j}^{s}\beta_{ij}x_{i}x_{j}+\varepsilon$$

The real friction surfacing process is carried out after the preparation of the mechtrode and substrate has been completed. The mechtrode is fastened on a collet, fixed on a rig. The layer in the moving table is made for fixation. The parameters for the system were loaded in the software manually. The final friction surfacing of the piece of work was then carried out. A few necessary time periods can be taken to form the mechtrode flash. Upon reaching the plastic deformation state, the metal continues to deposit on the substrate. With traverse speed, the table is moved along the length of the substrate to get the metal-coated. A specified duration of the deposit was added. The plate from the device is then disassembled. The rod diameter and the thickness of the coating will not be the same as the rotational force acting on the center of the rod and will not be dispersed to the edges sufficiently. It would then have a regular coating on one side and an inaccurate coating on the other side. The advancing side is known as the side that has a uniform coating, and the other side is known to be retreating. The mechtrode rotation and plate shift in the forward line is in the opposite direction, thereby achieving straight coating. The procedure is done by making up the contact between the substratum and mechtrode for a certain duration and then moving the disk. In addition, the 20 test trail is repeated for varying process parameters until the consumable rod is worn off.

After the friction surfacing process is finished, the workpiece is cut off into small parts for the further investigative process. The material is sliced into the coated region where it is properly bound to the material. Using the technique of wire cutting, the sample is cut to allow the exact center of the coated substrate. The coating dimensions and debonded patches can be assessed using image processing algorithms that incorporate image segmentation based on selected threshold levels of the image histogram.

Specimens were ground using coarse and fine grinding sheets, then polished with a 0.5 m diamond suspension for the final polish. To improve and disclose the microstructure, the cross-section was etched with a 4 percent nitric acid solution. An FEI Quanta FG200 high-resolution scanning electron microscope was used to examine the specimens.

## 3. Results and Discussion

The principal aim of friction surfacing is to deposit material through plasticization. The feasible parameter is selected such that the AISI316Ti alloy is coated without any defects, using EN8 medium carbon steel. Without loss of accuracy, in the RSM, the central composite architecture of the quadratic form seems useful in modeling the analytical model with the least number of trials. Friction surfacing was performed and the response to the feedback was recorded, as seen in Table 4, according to the experimental findings.

In terms of coded variables, the equation is used to make predictions about the response of each factor for given rates. The high factor rates are coded as +1, and the low factor values are coded as −1. When comparing factor coefficients for response characteristics such as coating width, coating thickness, and bond strength to represent the relative impact of the components, the coded equation derived from regression models was useful. Bonding coating dimensions were found on the parametric stages, and the grains were dynamically recrystallized on both the withdrawing and advancing sides due to rotational velocity [28]. The significant factor effects were obtained from the regression models for the output parameters to assess the influence of the experiment’s input parameters, following the research carried out by George Sahaya Nixon et al. [29]. The test was carried out on the basis of the regression model and individual coefficient model. The ANOVA tests were performed to show that the coefficient is important, and to reduce the reduction of the model. A typical regression model is suitable for fit if the R2 value is below 1. In accordance with Vijaya Kumar et al. [30], reduced quadratic form models for the coating thickness, coating width, and bond strength are set out in Table 5, Table 6 and Table 7. Model F-value is important for coating thickness (400.41), coating width (294.39), and bond strength (36.08). There is only a possibility that the model F-value may be greater by 0.01 percent which is due to noise occurrence. The values of Prob > F in Table 4, Table 5 and Table 6 suggest that the terms of the model are relevant with a value of 0.05, i.e., a confidence level of 95 percent. For the output responses, the combinations of input parameters such as A, B, C, AB, AC, BC, A^2^, B^2^, and C^2^ are therefore significant. The lack of fit values > 0.01 resembles not being meaningful. Because the terms of the model are not quite small, there is no need to enhance the model by model reduction. The number of model terms chosen is obviously within their limits. There is no major lack of fit for the coating thickness (4.61), coating width (25.88), and bond strength (68.42). Owing to the incidence of noise which is 5.95 percent for coating thickness, 0.14 percent for coating width, and 0.01 percent for bond strength, there is a probability that the F-lack values of fit will be significant.

The mathematical model of the responses with respect to the input parameters is given in Table 8 from which the experimental value can be compared with the predicted value for the coating thickness, coating width, and bond strength. The statistical model selected in this experimental investigation is significant, the physical significance of the entire experimentation lies in these three selected independent or control variables only, the mechanism exemplifies that, the maximum axial load provides maximum dispersion of the coating material, the more the load applied the more is the plastic deformation of the selected material. Under heavy loading conditions, the rubbing action between the mating surfaces increases not only heat but high surface energy. This drastic increase in surface energy and heat increases the rate of deposition and consequently the coating thickness.

Similarly the role of transverse speed, at high loading conditions if we keep the traverse speed between minimum to normal, the thrust force increases, this increase in thrust forces increases the coating thickness. At higher traverse speed, the uniformity in the thickness would be much disturbed. So, the parameter Traverse speed plays a significant role. If it is too high there would be likely damage to the mechatrode, which is not advisable, if the intention is to obtain a proper finish.

The role of rotational speed is the key in promoting the heat energy developed between the surfaces. The more heat energy developed, the more there would be the melting of the consumable mating member. If the axial load is high, there is a drastic increase in heat energy developed along with axial thrust, here, the coating width would increase keeping the thickness in check. At high rotational speeds, the axial forces must be moderate and the traverse speed must be minimum.

Given the aforementioned conditions, a good coating thickness would be achieved. So, it could be concluded that the selected three parameters are really significant to have better thickness and normal coating width at an appreciable surface finish. The number of experiments selected here provides sufficient information on the roles played by the individual control parameters over the dependent variables. The interactions between these independent or control variables are sufficient to justify the outputs of the experimentation.

The statistics obtained for response parameters such as R2, modified R2, and projected R2 are shown in Table 9. It was observed that the values are similar to unity, suggesting that the response model matches the experimental data obtained better. The R2 for coating thickness expected is 0.9732 which is in practical arrangement with the 0.9947 modified R2. Likewise, the projected R2 is obtained for coating width and bond strength as 0.9721 and 0.8738, which is in practical arrangement with the modified R2 of 0.9929 and 0.9432 as validated by Mostafapour et al. [31]. The difference between the modified R2 and the expected R2 is less than 0.2. The ratio of acceptable precision for coating thickness (74.7390), coating width (56.8458), and bond strength (21.1127) shows that the signal is sufficient and satisfactory.

**Figure 2:**
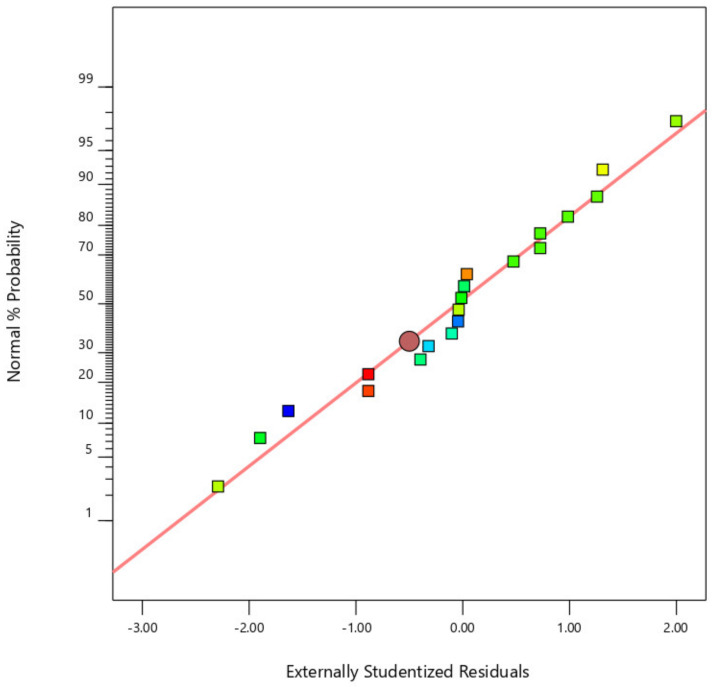
Normal plot of residuals for coating thickness (C_t_).

**Figure 3:**
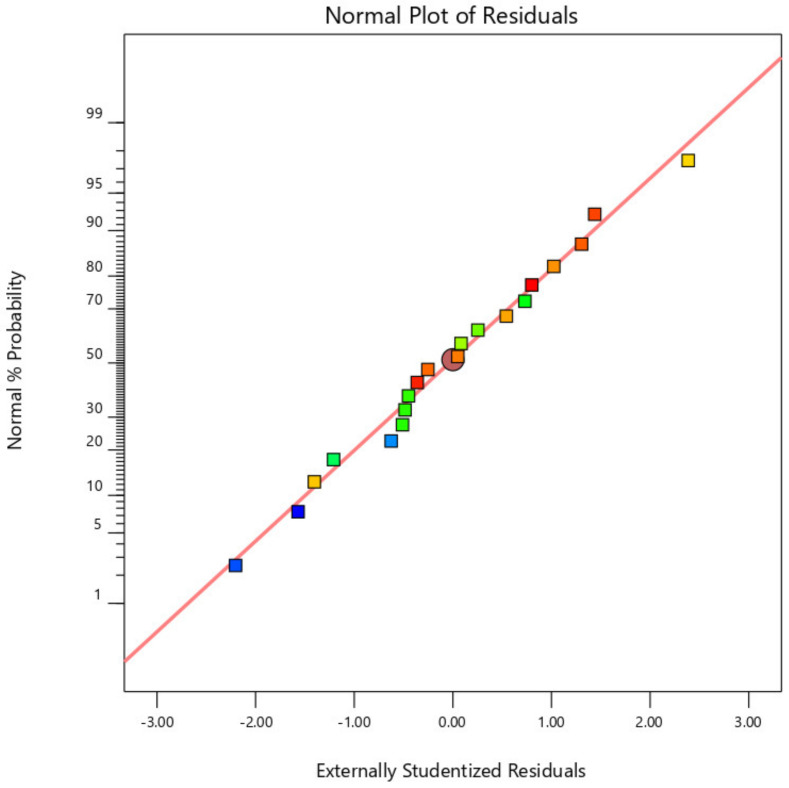
Normal plot of residuals for coating width (C_w_).

**Figure 4:**
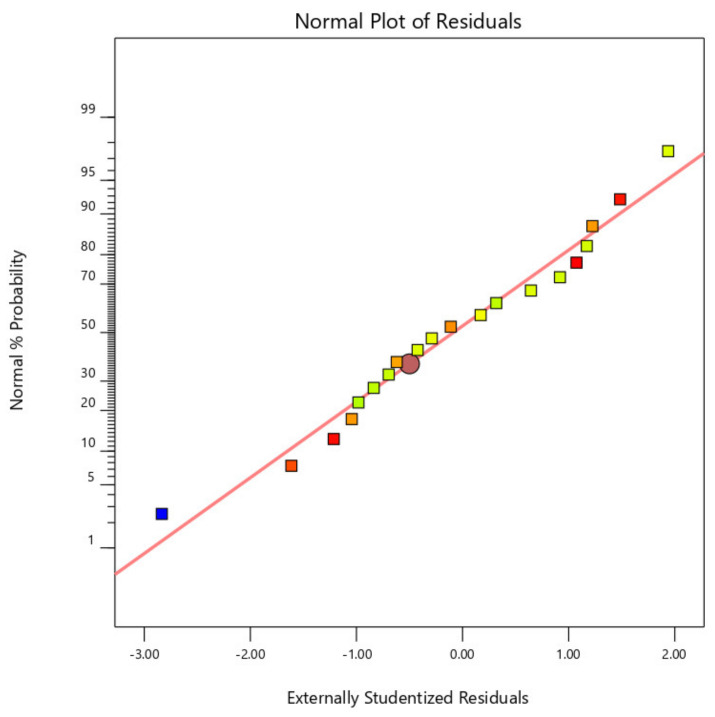
Normal plot of residuals for bond strength (B_s_).

Figure 2, Figure 3 and Figure 4 display the standard plot of residuals for coating thickness (C_t_), covering width (C_w_), and bond strength (B_s_). It is clear that the points on the standard plot are similar to the straight line and due to the expected R2 values of 0.9732 and 0.9721, there are no differences in the thickness and width of the coating. Some amount of deviations in the bond strength is observed due to the material being plasticized during the friction surfacing process. The predicted R2 for bond strength is lower than the coating thickness and coating width values, which is 0.8738, resulting in a deviation from the straight line of the normal plot due to which the percentage of error of the actual value with the predicted value will increase.

### 3.1. Impact of Process Parameters on Responses

**Figure 5:**
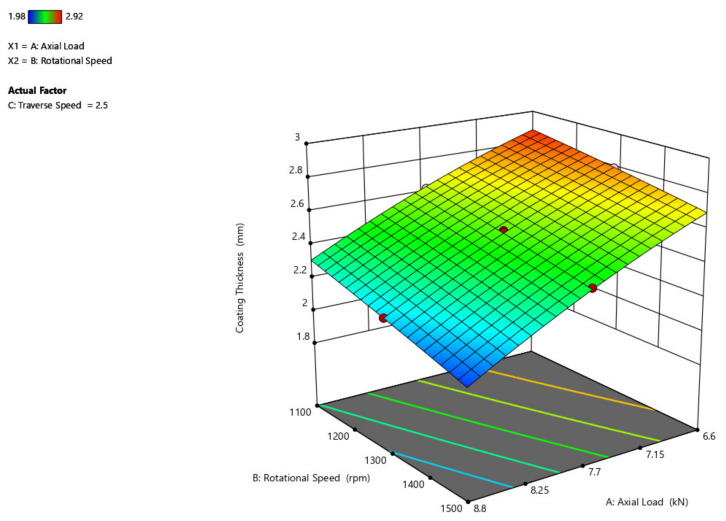
Interaction plot of axial load and rotational speed on coating thickness (C_t_).

Figure 5 shows that the increase in axial load reduces the coating thickness and that the change in rotational speed just slightly raises the coating thickness. This is due to the increase in heat generation resulting from the increase in axial load. The increase in coating thickness due to the rise in rotational velocity is due to the increase in torque due to friction between the mechtrode and the substratum, which causes the rate of deformation of consumable material to rise.

**Figure 6:**
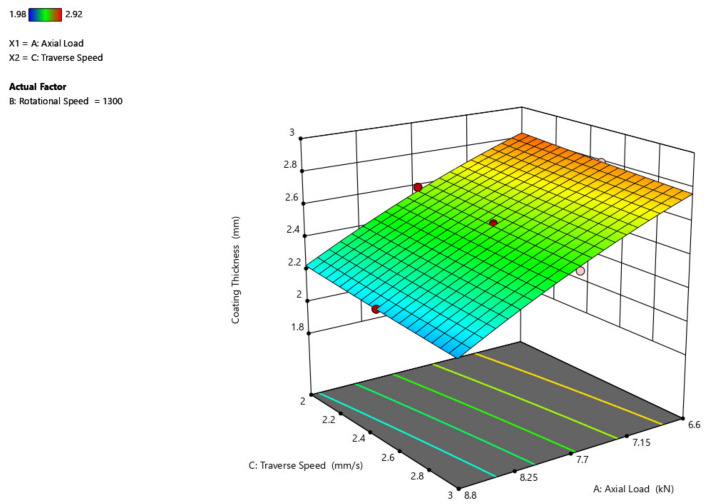
Interaction plot of traverse speed and axial load on coating thickness (C_t_).

In comparison to the axial load and rotational speed, the traverse speed has no detrimental influence on the thickness of the coating, as shown in Figure 6. Despite the increased traversal speed, the coating thickness stays constant [32]. When the traverse speed is maximum (3 mm/s) and the axial force is maximum (8.8 kN) the thickness of the coating would be minimal.

**Figure 7:**
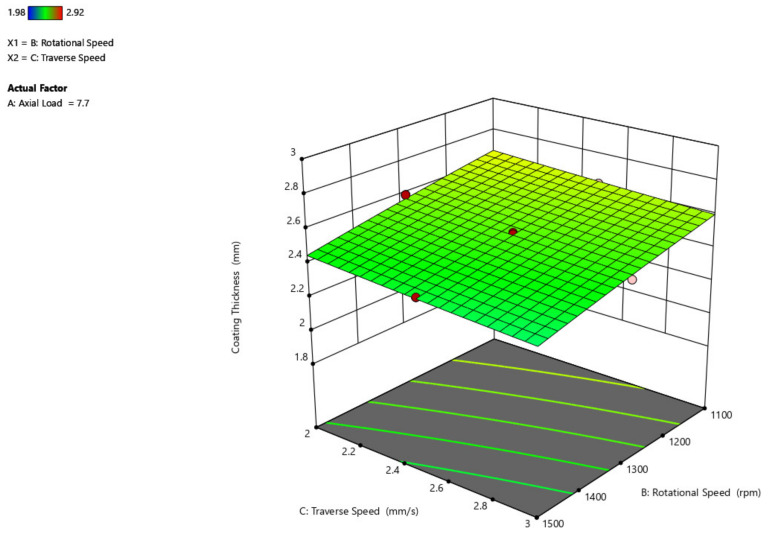
Interaction plot of traverse speed and rotational speed on coating thickness (C_t_).

The effect of rotational velocity and traverse speed over the thickness of the coating is shown in Figure 7. An increase in rotational speed from 1100 rpm to 1500 rpm decreases the thickness of the coating to some degree to around 0.2 mm. By increasing the traverse speed from 2 mm/s to 3 mm/s, the thickness of the coating remains constant without showing any difference because of the region of contact between the substrate and the mechtrode. This shows that when compared to the rotational speed, the traverse speed does not have any detrimental impact on the thickness of the coating, as its impact is minimal.

**Figure 8:**
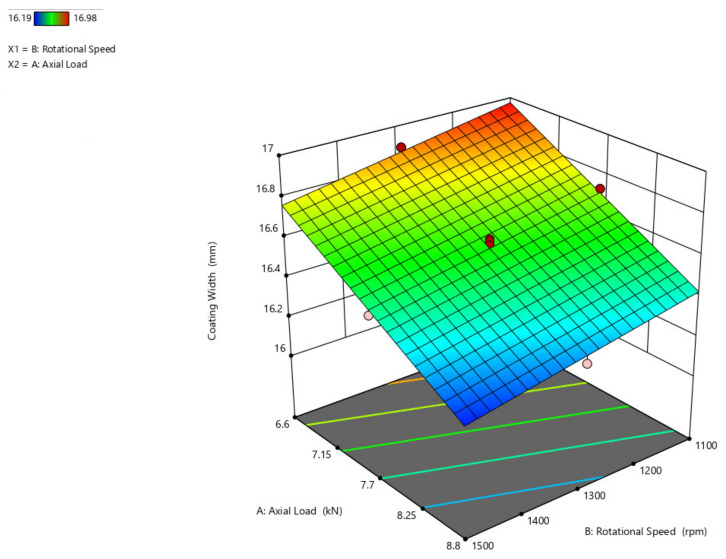
Interaction plot of rotational speed and axial load on coating width (C_w_).

The influence of rotating speed and axial load on coating width is shown in Figure 8. The coating width increases as the axial load increases from 6.6 kN to 8.8 kN with a range of 5 mm, and the coating width increases to a reduced point of 1 mm with the increasing rotational speed even.

**Figure 9:**
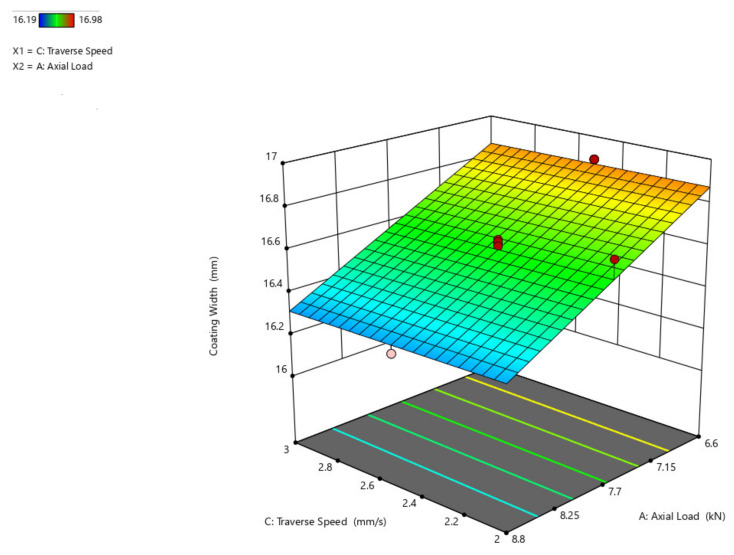
Interaction plot of axial load and traverse speed on coating width (C_w_).

The 3D surface plot is shown in Figure 9, showing the influence of axial load and traverse speed over coating width. If studied from the preceding figure, it is anticipated that as the axial load increases, the width of the coating grows and that when compared to the traverse speed, the width of the coating has little influence, where the width increases by a maximum of 1 mm.

**Figure 10:**
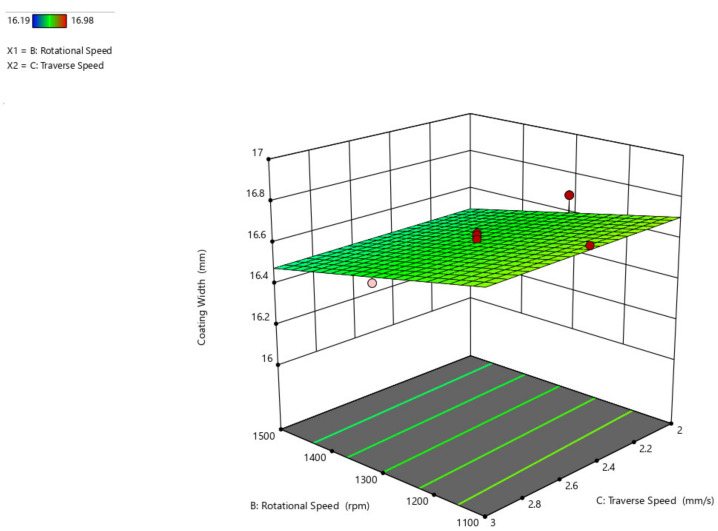
Interaction plot of traverse speed and rotational speed on coating width (C_w_).

Figure 10 depicts a three-dimensional graph of the response surface for coating width as a function of traversal speed and rotating speed of the parameter. When the rotating speed is increased, the coating width increases, but when the traverse speed is increased from 2 mm/s to 3 mm/s, the coating width remains nearly unchanged.

**Figure 11:**
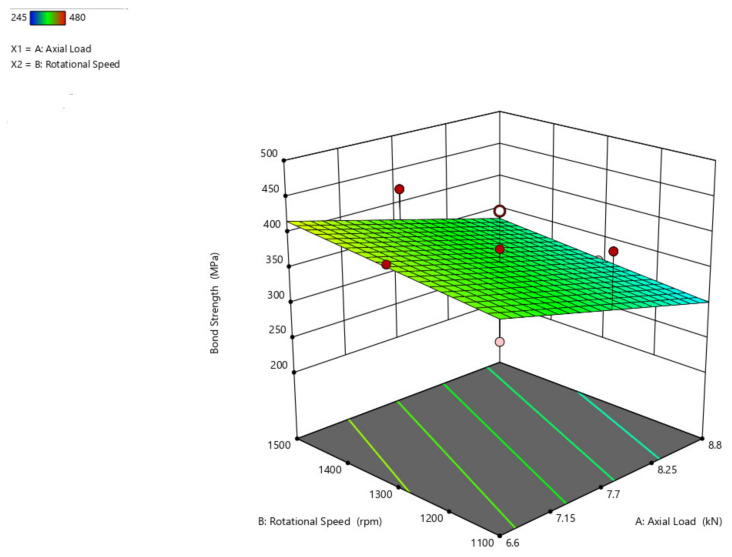
Interaction plot of axial load and rotational speed on bond strength (B_s_).

**Figure 12:**
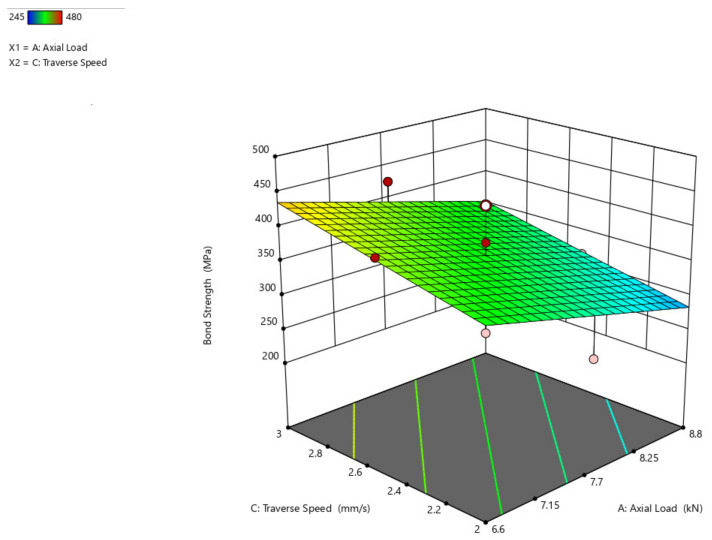
Interaction plot of axial load and traverse speed on bond strength (B_s_).

**Figure 13:**
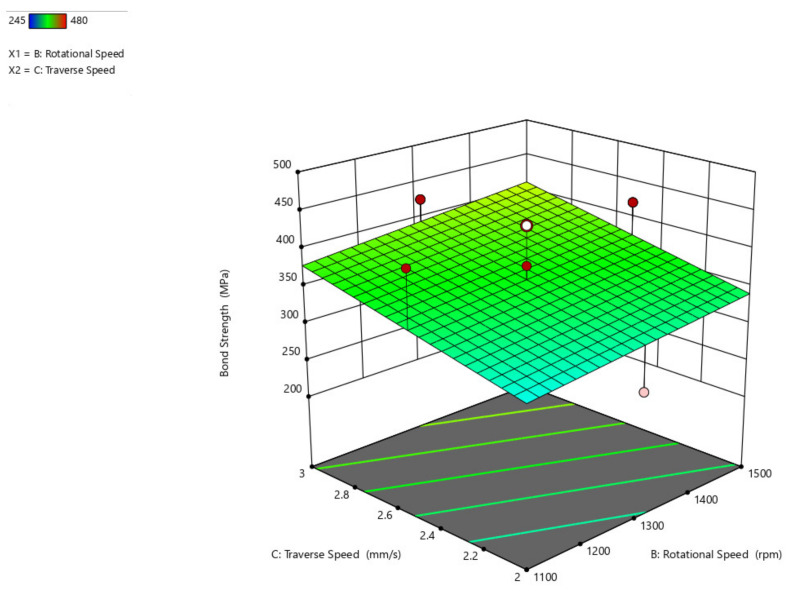
Interaction plot of traverse speed and rotational speed on bond strength (B_s_).

The bond strength interaction plot with respect to the axial load and rotational speed parameters (Figure 11), axial load and traverse speed (Figure 12), and rotational speed and traverse speed (Figure 13), is shown. There is an ascending peak in the values of the bond strength which ranges from 300 MPa to 425 MPa for an increase in the axial load from 6.6 kN to 8.8 kN and also has less effect on the rotational speed and traverse speed to the bond strength. As the rotational speed increases, the bond strength gradually diminishes.

From the response surface graph shown in Figure 12, it is concluded that there is an increase in axial load from 6.6 kN to 8.8 kN, a significant increase in bond strength from 300 MPa to almost 450 MPa, and a decrease in bond strength with an increase in traverse speed and slowly increases with an increase in traverse speed from 2.6 mm/s to 3 mm/s. When compared to axial load, the traverse speed has less impact on the bond strength.

As evident from previous figures and with respect to Figure 13, the rise in rotational speed and the decrease in traverse speed is thought to cause a drop in bond strength, resulting in strong bonding and an increase in bond strength.

### 3.2. Performance of an Additivity Test

**Figure 14:**
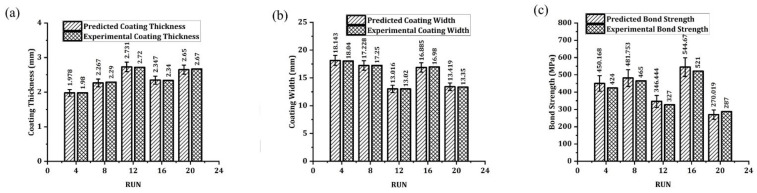
Additivity test results illustrating the predicted value and the experimental value for (**a**) coating thickness, (**b**) coating width, and (**c**) bond strength.

At a random interval of four runs, the parametric combinations taken from the experimental design are chosen to evaluate the variance in the compilation between the predicted value and the experimental coating thickness (C_t_) as shown in Figure 14a, coating width (C_w_) as shown in Figure 14b, and bond strength (B_s_) as shown in Figure 14c. The percentage of differences in coating width and coating thickness tends to be ±5 percent, although there is a difference of ±8 percent in the case of bond strength. This difference in bond strength is due to the material plasticization between the mechtode and the substrate. Through this study, the advantage that emerged helps us to predict the vital responses of coating width, coating thickness, and bond strength, during friction surfacing of AISI316Ti over EN8 alloy.

### 3.3. Analysis of Microstructure

**Figure 15:**
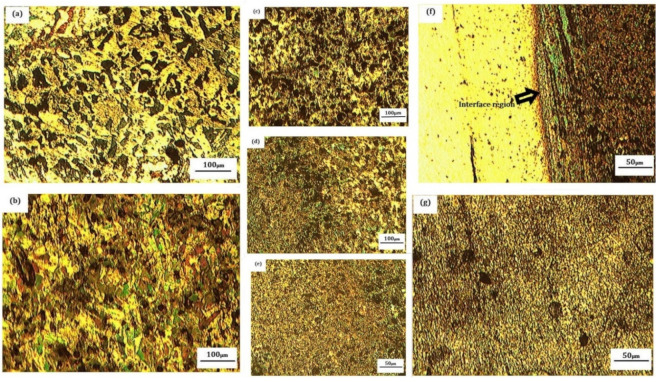
Microstructure analysis of friction surfacing AISI316Ti over EN8 alloy: (**a**) grain structure of the substrate; (**b,c**) grain structure of the mechtrode; (**d–g**) microstructure at the interface regions.

The micro-structure indicates inter-metallic mixing between the two base metals for the specimen, the micro-structure shows ferrite-pearlite grains moving towards the matrix, and subsequently, heat-affected zone (HAZ) showing small grains and deformed martensite to some degree. More strength was denoted due to an unfinished martensitic structure shown in Figure 15a–c The micro-structure shows uniform pearlite grains in the ferrite matrix [32]. The grains are elongated along the rolling direction. The Steel’s grain size is 25 to 30 microns. The pearlite grains are present with the ferrite grain boundaries and demonstrate the fractography in the base metals of the plate and the rod. Due to dynamic re-crystallization, a very fine-grained micro-structure is produced towards the process, as the base metals perform a thermo-mechanical phenomenon. The fractographic study reveals that austenite, delta ferrite, and a trace amount of chromium-and-precipitate contain pearlite ferrite matrix, indicating a tight link between the specimens [7]. Figure 15d shows the fusion over HAZ in the substrate and the base metal that is not thermally affected. The HAZ shows very good re-crystallization, with a scale of between 8 and 10 microns. This clearly shows that the two metals are closely bonded. It was denoted that better strength was bonded to the mixing of these materials in the intermingling field. This ensures that the greater thermal effect was generated when combined with other steel matrices in the parental process of friction surfacing over AISI316Ti. Similarly, the cohesion matrix between AISI 316Ti and EN8 was lower than other steel types [33]. In addition, plasticity due to thermal effect results in complete austenitization and dynamic re-crystallization [34]. It has been noted that the dimensions of the coating might depend on the parametric phase and on the mechtrode’s rotating motion, in addition to the grains. Figure 15e shows the maximum region affected by heat near the weld zone with fine pearlite grains in ferrite. Figure 15f displays the interface region for the welded austenitic stainless steel and the low carbon steel parent metal [35]. The fusion zone is without discontinuity at the center. Figure 15g shows the stainless steel welded zone with fine austenite grains that are in the fine dendritic pattern [36].

### 3.4. Scanning Electron Microscope (SEM) Analysis

**Figure 16:**
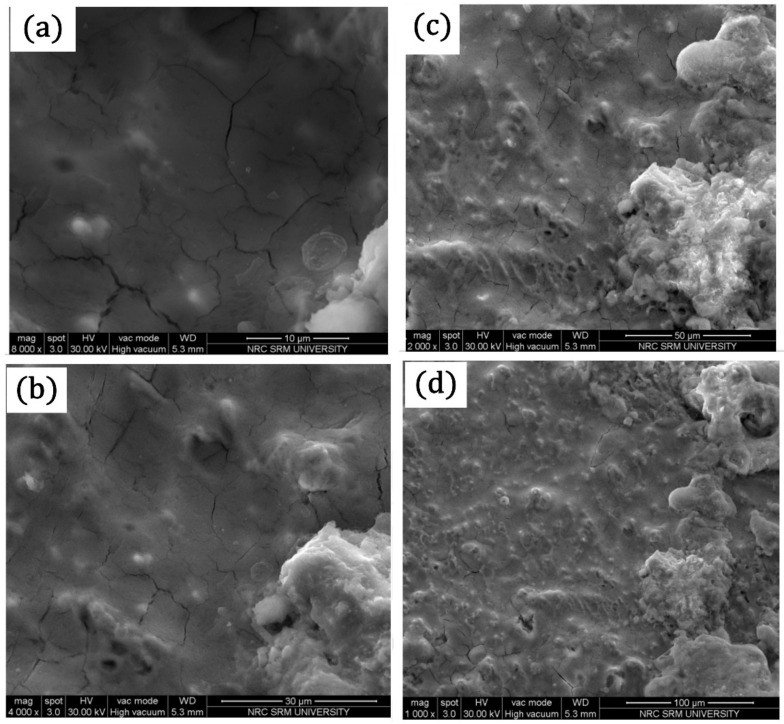

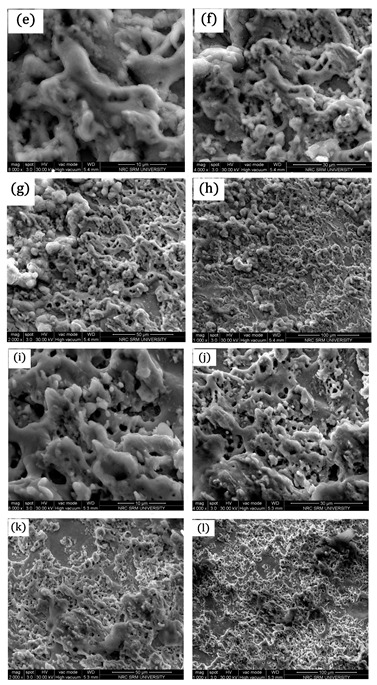
Scanning electron microscopic image analysis of the friction surfacing of AISI316 over EN8 alloy (**a**–**d**) interface region, (**e**–**h**) mechtrode surface, and (**i**–**l**) substrate surface.

The microstructure SEM study carried out using FEI Quanta FG200 HRSEM shown in Figure 16a–j, as obtained, indicates the specific magnification of AISI316Ti friction coated coatings over EN8 steel. As the optical microscopy did not foresee the secondary components, SEM images were taken out to know the dissolution of dissimilar materials. Large ferrites appeared during the solidification process which precipitated from the austenitic phase due to the very high alloy composition of EN8 steel. Dimensions of tool steel’s primary ferrite range between 5 μm and 10 μm in diameter. It is due to high deformation during the surfacing phase of the friction. Because of the complex re-crystallization, the grain sizes were reduced to 2–3 μm when the metallic materials were subjected to extreme plastic deformation [37]. The grain coarsening occurs due to the heat produced in the high reduction hot working cycle, which causes high dislocation density and numerous stacking faults due to very high deformation and combined effect of heat and plastic deformation [38], which causes high dislocation density and numerous stacking faults. Due to the phase transition from BCC to FCC, the EN8 steel requires partial austenitization and often provided coarse and heterogeneously scattered carbides in solidified conditions.

The mechtrode metal micro-structure is distinctly austenitic. AISI 316Ti’s micro-structure includes ferrite grains that range from 40–70 μm in diameter with randomly dispersed heterogeneous injection (Figure 16a–d,h–j). These appearances were found earlier on to any surface formation in as-prepared specimens. According to earlier research on AISI 316Ti [39], a series of EDX line inspections discovered the composition of these inclusions to be predominantly titanium, copper, carbon, and zinc, with most inclusions being TiC. Both materials typically show a distinctive austenitic micro-structure and equally unevenly formed Ti(C) (cubic) crystals, due to the addition of Ti to the mixed stainless steel proportion. The occurrence of grain boundary phases after mechtrode process has not been established. The surface grains formed could be created by the mechtrode method. Experiential micro-structures contain titanium carbide (Figure 16e–g). It (Ti) is cubic form, carbides (Ti, Cr) are condensed into sheets. The surface of steel was completely covered by the coating process and formed a micro layer on it. This can increase metal hardness, shape a protective coating, corrosion resistivity, wear resistivity, and tear resistivity for further applications. Titanium was found to be a more cathodic than anodic metal such as copper and zinc. If less copper (thin-film copper oxide) and more electron releasing active metal zinc are in mechtrode, this composition regulates corrosion on the metal surface and the steel surface is unaffected. The Ti composition simultaneously regulated metal reactivity and light absorption. Easy weight loss approach in an acidic environment. Figure 16a–c proved the resistivity to corrosion of metals. The specific concentration of the evaporative hydrochloric acid solution was used to check the corrosion activity of mechtrode. However, the weight losses detected are very low and are between 6 and 9 mg. Hence, this proved the steel’s corrosion resistivity and demonstrated strong resistance [40,41,42,43,44,45,46].

## 4. Conclusions

Based on process rates of process parameters using AISI316Ti over EN8’s response surface methodology for friction surfacing, the number of experiments is expected. The output parameters related to the process parameters are derived from the response surface analysis methods.

For coating width, coating thickness, and bond strength, and to aid in the evaluation of the link between the parameters of the input method, mathematical models have been devised and the response parameters at a confidence level of 95 percent to achieve maximum coating width and minimum coating thickness.

It was found that the parameters of the chosen process decide the bonding geometry. The thickness of the coating decreases as the axial load increases, which has an effect on the direction of traverse and speed.

Increasing the impact with the axial load and rotation speed gives greater width of the coating and equal bonding strength. These input parameters also have a positive influence on the geometry of bonding and bonding strength. Traverse speed has little control over the thickness of the coating and the bond strength.

The optimum process parameter rates were attained at an axial load of 7.7 kN, a rotational speed of 1300 rpm, and traverse speed of 2.5 mm/s. The optimal performance for the above process parameter was obtained to be coating thickness (C_t_) of 2.52373 mm, coating width (C_w_) of 15.5438 mm, and bond strength (B_s_) of 362.364 MPa.

SEM research was performed on the samples in order to understand the microstructure effect of the method on the substrate base, interface, and coating regions. It is well known from the SEM images that the key mechanism controlling the friction surfacing process is dynamic re-crystallization.

## Figures and Tables

**Table 1:** Chemical composition of mechtrode AISI316Ti.

Material	C	Si	Mn	S	P	Cr	Mo	Ni	Ti	Fe
Composition in %	0.08	0.75	2.0	0.03	0.045	17.5	2.46	12	0.70	Remainder

**Table 2:** Chemical composition of substrate EN8.

Material	C	Si	Mn	S	P	Cr	Ni	Fe
Composition in %	0.42	0.20	0.65	0.015	0.026	0.01	0.01	Remainder

**Table 3:** Parameters and their corresponding levels.

Levels	Axial Load in kN (A)	Rotational Speed in rpm (B)	Traverse Speed in mm/s (C)
Level 1	6.6	1100	2.0
Level 2	7.7	1300	2.5
Level 3	8.8	1500	3.0

**Table 4:** Responses for the various parameter levels.

Std	Run	Factor 1: A: Axial Load in kN	Factor 2: B: Rotational Speed in rpm	Factor 3: C: Traverse Speed in mm/s	Response 1: Coating Thickness in mm	Response 2: Coating Width in mm	Response 3: Bond Strength in MPa
12	1	7.7	1500	2.5	2.41	16.17	362
1	2	6.6	1100	2	2.92	12.26	352
20	3	7.7	1300	2.5	2.53	15.58	361
8	4	8.8	1500	3	1.98	18.04	424
19	5	7.7	1300	2.5	2.51	15.62	351
9	6	6.6	1300	2.5	2.79	12.7	315
4	7	8.8	1500	2	2.09	17.86	539
6	8	8.8	1100	3	2.29	17.25	465
15	9	7.7	1300	2.5	2.54	15.51	354
10	10	8.8	1300	2.5	2.18	17.49	455
14	11	7.7	1300	3	2.46	15.92	374
3	12	6.6	1500	2	2.72	13.02	327
16	13	7.7	1300	2.5	2.52	15.6	352
18	14	7.7	1300	2.5	2.53	15.55	353
13	15	7.7	1300	2	2.59	15.16	408
2	16	8.8	1100	2	2.34	16.98	521
5	17	6.6	1100	3	2.86	12.59	324
17	18	7.7	1300	2.5	2.52	15.54	355
11	19	7.7	1100	2.5	2.62	14.85	405
7	20	6.6	1500	3	2.67	13.35	287

**Table 5:** ANOVA for quadratic model—Response 1: Coating thickness (C_t_).

Source	Sum of Squares	Degrees of Freedom	Mean Square	F-Value	*p*-Value
Model	1.11	9	0.1235	400.41	<0.0001
A-Axial Load (kN)	0.9486	1	0.9486	3076.59	<0.0001
B-Rotational Speed (rpm)	0.1346	1	0.1346	436.40	<0.0001
C-Traverse Speed (mm/s)	0.0160	1	0.0160	51.89	<0.0001
AB	0.0036	1	0.0036	11.72	0.0065
AC	0.0003	1	0.0003	1.01	0.3378
BC	0.0003	1	0.0003	1.01	0.3378
A²	0.0037	1	0.0037	12.09	0.0060
B²	0.0001	1	0.0001	0.4146	0.5341
C²	0.0000	1	0.0000	0.0903	0.7700
Residual	0.0031	10	0.0003		
Lack of Fit	0.0025	5	0.0005	4.61	0.0595
Pure Error	0.0006	5	0.0001		
Cor Total	1.11	19			

**Table 6:** ANOVA for quadratic model—Response 2: Coating width (C_w_).

Source	Sum of Squares	Degrees of Freedom	Mean Square	F-Value	*p*-Value
Model	59.35	9	6.59	294.39	<0.0001
A-Axial Load (kN)	56.17	1	56.17	2507.54	<0.0001
B-Rotational Speed (rpm)	2.03	1	2.03	90.80	<0.0001
C-Traverse Speed (mm/s)	0.3497	1	0.3497	15.61	0.0027
AB	0.0028	1	0.0028	0.1256	0.7304
AC	0.0055	1	0.0055	0.2461	0.6306
BC	0.0010	1	0.0010	0.0452	0.8359
A²	0.4726	1	0.4726	21.10	0.0010
B²	5.682 × 10^−07^	1	5.682 × 10^−7^	0.0000	0.9961
C²	0.0026	1	0.0026	0.1139	0.7428
Residual	0.2240	10	0.0224		
Lack of Fit	0.2157	5	0.0431	25.88	0.0014
Pure Error	0.0083	5	0.0017		
Cor Total	59.57	19			

**Table 7:** ANOVA for quadratic model—Response 3: Bond strength (B_s_).

Source	Sum of Squares	Degrees of Freedom	Mean Square	F-Value	*p*-Value
Model	52,590.25	9	5843.36	36.08	<0.0001
A-Axial Load (kN)	45,832.90	1	45,832.90	282.96	<0.0001
B-Rotational Speed (rpm)	3763.60	1	3763.60	23.24	0.0007
C-Traverse Speed (mm/s)	624.10	1	624.10	3.85	0.0781
AB	21.13	1	21.13	0.1304	0.7255
AC	1.13	1	1.13	0.0069	0.9352
BC	1.13	1	1.13	0.0069	0.9352
A²	158.46	1	158.46	0.9783	0.3459
B²	180.02	1	180.02	1.11	0.3166
C²	252.96	1	252.96	1.56	0.2399
Residual	1619.75	10	161.98		
Lack of Fit	1596.42	5	319.28	68.42	0.0001
Pure Error	23.33	5	4.67		
Cor Total	54,210.00	19			

**Table 8:** Regression model equation for the responses.

Responses	Regression Model
Coating thickness (Ct)	$+2.23236+\left(0.342572\times A\right)+\left(0.000763\times B\right)+\left(0.025114\times C\right)-\left(0.00097\times AB\right)-\left(0.011364\times AC\right)-\left(0.000063\times BC\right)-\left(0.030428\times A^{2}\right)-\left(1.70455E^{-07}\times B^{2}\right)+\left(0.01727\times C^{2}\right)$
Coating width (Cw)	$-24.87609+\left(7.43910\times A\right)+\left(0.001850\times B\right)+\left(0.278659\times C\right)-\left(0.000085\times AB\right)-\left(0.047727\times AC\right)-\left(0.000113\times BC\right)-\left(0.342600\times A^{2}\right)-\left(1.13636E^{-08}\times B^{2}\right)+\left(0.121818\times C^{2}\right)$
Bond strength (Bs)	$+908.38182-\left(23.7593\times B^{2}\right)-\left(0.556659\times C^{2}\right)-\left(197.49318\times D^{2}\right)+\left(0.007386\times B^{2}\times C^{2}\right)-\left(0.681818\times B^{2}\times D^{2}\right)-\left(0.0375\times C^{2}\times D^{2}\right)+\left(6.27348\times B^{2}\times B^{2}\right)+\left(0.000202\times C^{2}\times C^{2}\right)+\left(36.36364\times D^{2}\times D^{2}\right)$

**Table 9:** Regression statistics for coating thickness (C_t_), coating width (C_w_), and bond strength (B_s_).

Statistics	Coating Thickness (C_t_)	Coating Width (C_w_)	Bond Strength (B_s_)
R²	0.9972	0.9962	0.9701
Adjusted R²	0.9947	0.9929	0.9432
Predicted R²	0.9732	0.9721	0.8738
Adeq Precision	74.7390	56.8458	21.1127

## Data Availability

Not applicable.
